# Adhesive Colonic Obstruction After Esophageal Reconstruction: An Uncommon Late Complication

**DOI:** 10.7759/cureus.97760

**Published:** 2025-11-25

**Authors:** Hugo Pais Moreira, Victor Viegas, Hugo Pereira, João Varanda, Hugo Louro, Bela Pereira

**Affiliations:** 1 General Surgery, Unidade Local de Saúde Gaia/Espinho, Porto, PRT

**Keywords:** adhesiolysis, caustic ingestion injury, colon interposition, esophageal reconstruction, late surgical complication

## Abstract

Adhesive colonic obstruction is a rare but clinically significant late complication following esophageal reconstruction with colon interposition. This case report describes a 68-year-old woman with a history of caustic esophageal injury and colonic interposition, presenting 12 years postoperatively with acute intolerance to oral intake and thoracic colonic distension. Imaging and endoscopy confirmed mechanical obstruction due to an adhesion. Surgical adhesiolysis restored conduit patency, resulting in uneventful recovery. This case highlights the importance of long-term surveillance and prompt multidisciplinary management for late mechanical complications in colonic interposition patients, as late morbidity - including obstruction, redundancy, and stricture - can occur years after initial surgery and may require surgical intervention to preserve conduit function and patient quality of life.

## Introduction

Colonic interposition is a well-established technique for esophageal reconstruction when the stomach is unavailable, most commonly due to prior gastrectomy, extensive neoplastic involvement, or severe caustic injury. The procedure restores alimentary continuity using a segment of colon, with conduit selection - right, left, or transverse colon - guided by vascular anatomy, required length, and patient-specific factors. The left colon is often favored for its consistent blood supply, though the right colon may be selected for longer grafts or specific anatomical considerations [1-12].

Routing options for the colonic conduit include the posterior mediastinum, retrosternal, or subcutaneous pathways [2-9]. The posterior mediastinal route is generally preferred for optimal functional outcomes, but retrosternal or subcutaneous routes may be indicated in cases of prior mediastinal pathology or advanced malignancy [13-14]. Technical refinements such as microvascular "supercharging" and intraoperative fluorescence imaging have improved graft perfusion and reduced ischemic complications [15].

Despite advances, late complications remain clinically significant. These include anastomotic stricture, conduit redundancy, reflux, adhesive obstruction, and neoplasia. Anastomotic stricture may occur at the pharyngocolonic or esophagocolonic junction, often managed with endoscopic dilation. Conduit redundancy can result in dysphagia or regurgitation, sometimes necessitating surgical revision [1,3,5-6].Reflux and altered bowel motility are common functional sequelae [8]. Adhesive colonic obstruction, although rare, is a late mechanical complication that can present years after reconstruction and requires prompt recognition and multidisciplinary management. Additionally, neoplastic transformation within the conduit, though infrequent, underscores the need for long-term surveillance [12-16].

This article focuses on adhesive colonic obstruction as a late complication, highlighting its pathophysiology, clinical presentation, diagnostic approach, and management within the context of contemporary esophageal reconstruction practice.

## Case presentation

A 68-year-old woman presented to the emergency department with acute intolerance to oral intake, progressive dysphagia, and marked thoracic distension, suggestive of a high-grade obstruction of the upper gastrointestinal tract. Her past medical history was significant for a suicide attempt by caustic soda ingestion, which caused extensive esophageal and gastric necrosis. She was 56 years old at the time and had no prior surgical history. She subsequently underwent esophagectomy and gastrectomy with colonic interposition. Because the surgeons believed the conduit had poor blood supply, they opted for a subcutaneous route instead of a mediastinal or retrosternal one, aiming to better monitor and manage any potential complications arising from this limited vascularization. She remained asymptomatic for several years until the current episode.

**Figure 1:**
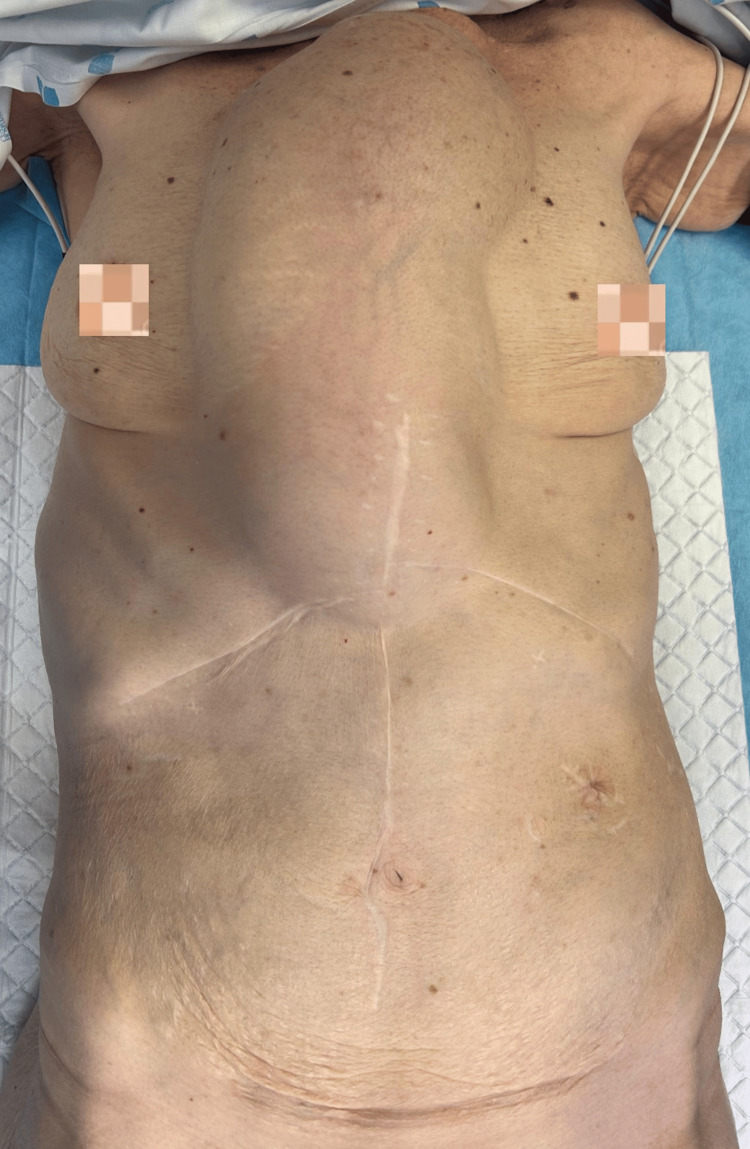
Distended thoracoabdominal colonic conduit at admission to the emergency department Initial chest and abdominal imaging at emergency presentation revealing pronounced dilatation of the interposed colonic conduit, consistent with acute obstruction.

On physical examination, she exhibited signs of proximal digestive obstruction - including pronounced thoracic distension, abdominal tenderness (Figure 1), and reduced bowel sounds with stable vital signs.

Laboratory tests were largely unremarkable except for mild dehydration. Relevant laboratory values are summarized in Table 1.

**Table 1:** Laboratory findings at presentation

Test	Patient Value	Reference Range	Interpretation
Hemoglobin	13.2 g/dL	12–16 g/dL	Normal
White Blood Cells	8.5 ×10³/µL	4–10 ×10³/µL	Normal
Sodium	145 mEq/L	135–145 mEq/L	Upper limit; consistent with mild dehydration
Potassium	4.0 mEq/L	3.5–5.0 mEq/L	Normal
Creatinine	1.1 mg/dL	0.6–1.2 mg/dL	Normal
Urea	45 mg/dL	15–40 mg/dL	Mildly elevated; consistent with dehydration
Lactate	1.8 mmol/L	0.5–2.2 mmol/L	Normal; no evidence of ischemia
Arterial pH	7.38	7.35–7.45	Normal
pCO₂	38 mmHg	35–45 mmHg	Normal
HCO₃⁻	24 mEq/L	22–26 mEq/L	Normal
C-Reactive Protein	1.2 mg/dL	<5 mg/dL	Normal

**Figure 2:**
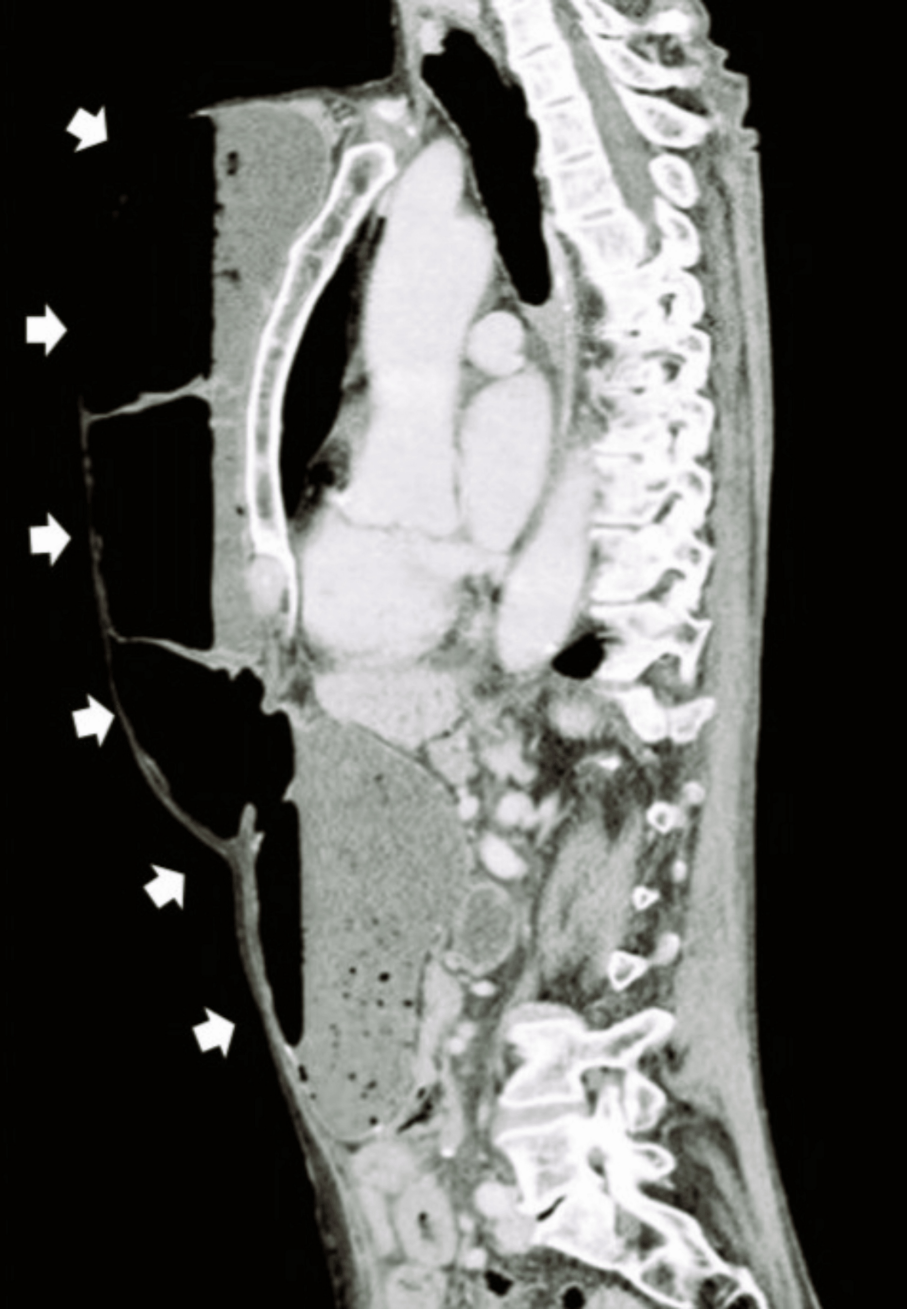
Sagittal Thoracoabdominal CT - Marked Neoesophageal Distension Contrast-enhanced thoracoabdominal CT showing significant distension of the interposed colonic conduit with no signs of perforation or ischemia, suggestive of mechanical obstruction, as indicated by the white arrows.

**Figure 3:**
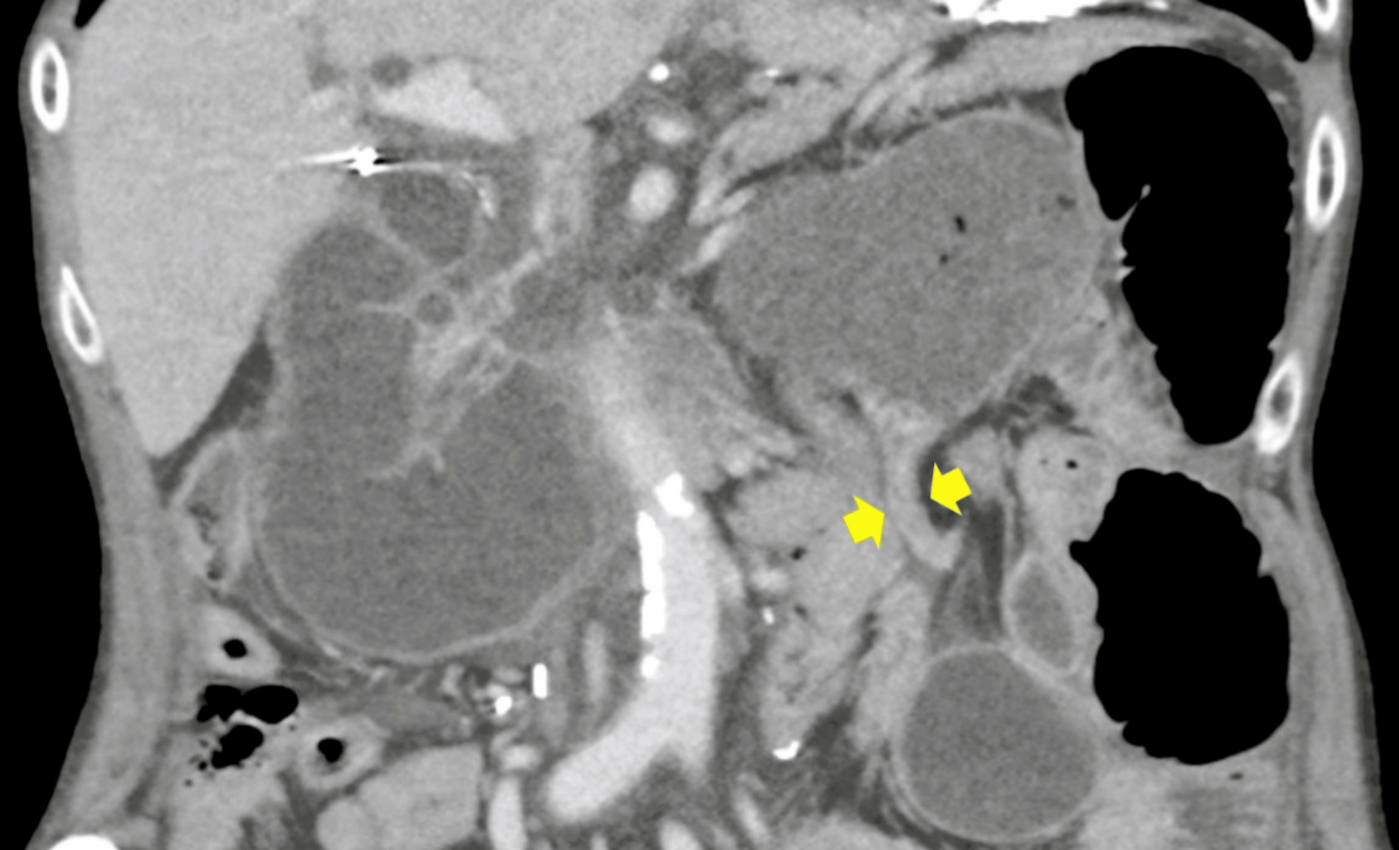
Transition Point Causing Obstruction of the Colonic Conduit Contrast-enhanced thoracoabdominal CT showing marked distension of the alimentary limb, with a transition point identified (yellow arrows), likely related to an adhesion in the context of the patient’s prior surgical history.

Contrast-enhanced computed tomography (CT) of the thorax and abdomen revealed marked distension of the entire neoesophagus up to the alimentary limb (Figure 2), where a transition point was identified, likely related to an adhesion, considering the patient’s prior surgical history (Figure 3). No evidence of perforation or ischemia was observed, suggesting a mechanical obstruction rather than a motility disorder.

**Figure 4:**
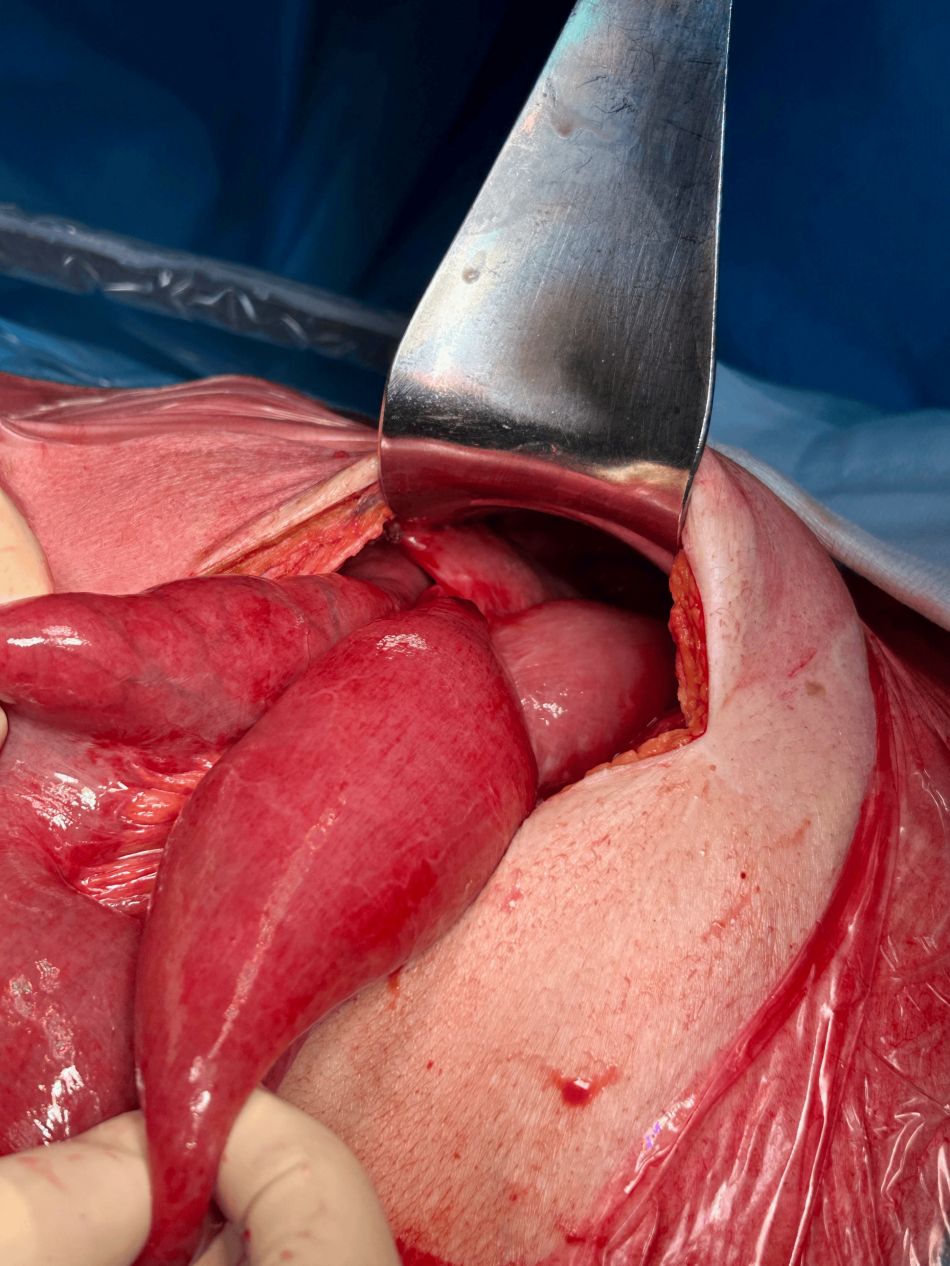
Marked Distension of the Alimentary Limb up to the Roux-en-Y Anastomosis Intraoperative inspection revealed marked distension of the alimentary limb up to the Roux-en-Y anastomosis.

Given the acute presentation and risk of conduit compromise, the patient was taken for urgent exploratory laparotomy. A midline supra- and infraumbilical laparotomy was performed. Intraoperatively, inspection revealed marked distension of the alimentary limb up to the Roux-en-Y anastomosis, caused by a dense adhesion (Figure 4). The adhesion band was carefully lysed, followed by decompression of the neoesophagus. The colonic vascular supply was preserved, and no additional strictures or lesions were observed.

**Figure 5:**
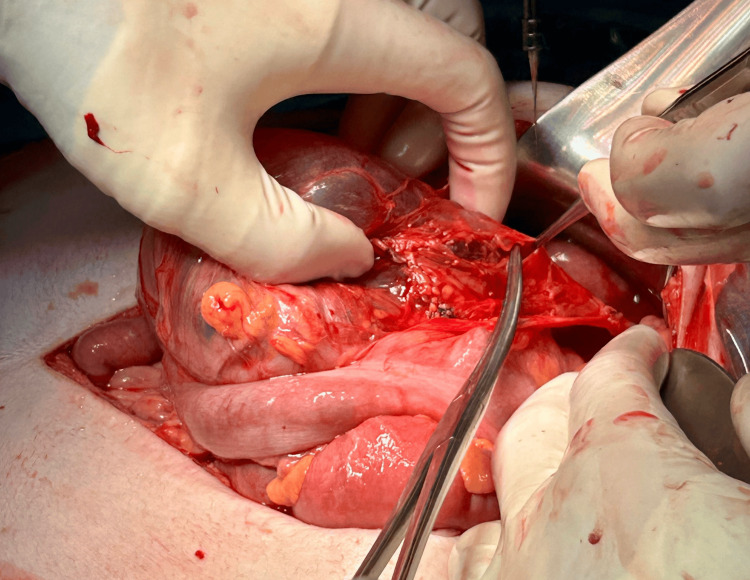
Intraoperative View of Adhesion Leading to Alimentary Limb Obstruction Intraoperative view showing a dense adhesion causing obstruction of the alimentary limb up to the Roux-en-Y anastomosis, successfully lysed to restore neoesophageal continuity.

Intraoperatively, a dense adhesion band was identified, causing kinking and functional obstruction of the interposed colon. The adhesion was meticulously divided, restoring luminal patency while preserving the colonic vascular supply. No additional strictures or lesions were observed (Figure 5).

The postoperative course was uneventful. The patient was initially maintained on parenteral fluids, followed by a gradual reintroduction of oral intake. By postoperative day 5, she was tolerating soft solids, and by day 7, she resumed a normal diet. She was discharged with recommendations for routine long-term follow-up to monitor for potential late complications, including adhesive obstruction or anastomotic stricture.

This case underscores the importance of recognizing late mechanical complications after colonic interposition, even many years after initial reconstruction. Although adhesive obstruction is uncommon, prompt diagnosis and timely surgical management are crucial to prevent severe morbidity and to preserve the reconstructed gastrointestinal continuity. The case also highlights the value of long-term surveillance in patients who have undergone complex esophageal reconstruction for caustic injuries.

Ethics statement

Written informed consent was obtained from the patient for publication of this case and the accompanying images. The study was conducted in accordance with institutional ethical standards and the principles of the Declaration of Helsinki.

## Discussion

Adhesive colonic obstruction is a rare but clinically significant late complication following esophageal reconstruction with colon interposition, most often performed in patients with caustic injury or when gastric pull-up is contraindicated due to prior surgery or poor vascular supply [12]. The left colon is generally preferred for its reliable marginal arterial arcade and favorable functional adaptation, but both right and transverse colon segments are used depending on patient anatomy and conduit length requirements [17-18].

Preoperative planning is critical and should include detailed vascular mapping, assessment of comorbidities, and careful selection of the conduit route (posterior mediastinal or retrosternal), as these factors influence both early and late outcomes [4]. Technical refinements such as meticulous dissection of the marginal artery and microvascular "supercharging" to cervical vessels have been shown to reduce anastomotic stricture rates and improve conduit viability, with lower rates of early complications and shorter hospital stays in patients receiving supercharged grafts [7-9].

Complications after colon interposition are classically divided into early and late [17]. Early events include graft necrosis, anastomotic leak, fistula formation, and perioperative mortality, with graft necrosis being the most feared due to its association with inadequate vascular supply or technical errors. Prevention relies on intraoperative perfusion assessment (e.g., indocyanine green fluorescence) and careful vascular management [1,12]. Early mortality in experienced centers remains low, typically 2-5% [12,17-21].

Late complications are more diverse and include anastomotic stricture (up to 36% of patients), conduit redundancy, reflux (bile or acid), adhesive obstruction, and, rarely, neoplasia within the colonic conduit [17]. Risk factors for late events include early reconstruction (<6 months after injury), insufficient enlargement of the thoracic inlet, emergency tracheotomy, and technical challenges during conduit mobilization [1-2,4,12,17]. Strictures are usually managed endoscopically, with surgery reserved for refractory cases [19-20]. Redundancy and reflux can severely impact swallowing and quality of life, sometimes necessitating segmental resection or diversion [21-24,25]. Adenocarcinoma may develop in long-standing colonic grafts, underscoring the need for long-term endoscopic surveillance [17,20,22,25].

Adhesive obstruction, while uncommon, is particularly challenging due to its delayed presentation and potential for significant morbidity. It typically results from fibrous band formation that kinks or compresses the conduit, leading to mechanical blockage. Factors influencing adhesion development include the initial surgical technique, postoperative inflammation, and individual healing characteristics. The incidence of adhesive bowel obstruction is higher after lower gastrointestinal tract surgery and in patients with severe adhesions at the operative site. Although the overall risk is low, the consequences can be severe, including conduit compromise and loss of alimentary continuity [17,22-24].

Diagnosis depends on a high index of suspicion, especially in patients presenting years after reconstruction with acute intolerance to oral intake, thoracic colonic distension, or intermittent dysphagia. Cross-sectional imaging (CT or MRI) and endoscopy are essential for distinguishing adhesive obstruction from other causes of late conduit failure such as stricture, redundancy, or neoplasia. Imaging may reveal segmental distension, abrupt transition points, or evidence of extrinsic compression, while endoscopy can confirm luminal patency and exclude mucosal pathology [17,19-20,22].

Management is dictated by the severity and chronicity of symptoms. Initial conservative measures (fluid resuscitation, nasogastric decompression, bowel rest) may be attempted in select cases, but surgical adhesiolysis is often required for definitive treatment of mechanical obstruction [3,19-21]. Revision surgery should be tailored to the underlying pathology, with segmental resection or stricturoplasty reserved for cases of redundancy or refractory stricture. Outcomes following successful intervention are generally favorable, with most patients regaining satisfactory alimentary function and quality of life [22-23,25].

Long-term follow-up is essential, as late complications may arise decades after reconstruction. Multidisciplinary management - including thoracic surgery, gastroenterology, and radiology - is critical for prompt diagnosis and intervention [19]. Future research should focus on identifying modifiable risk factors, optimizing surgical technique, and developing preventive strategies to minimize the incidence of adhesive obstruction and other late conduit failures [21,24].

In summary, adhesive colonic obstruction is a rare but important cause of late morbidity after esophageal reconstruction with colon interposition. Early recognition and prompt surgical management are critical to preserving conduit viability and patient quality of life [17,19,22-24].

## Conclusions

Adhesive colonic obstruction, although rare, should be considered in any patient with a colonic interposition who presents with obstructive symptoms years after surgery. Early recognition and timely surgical intervention are crucial to prevent graft compromise and reduce morbidity.

Colon interposition remains a valuable reconstructive option for selected patients with complex esophageal injury, offering acceptable long-term outcomes when performed with meticulous technique and followed by structured, multidisciplinary surveillance. Advances in vascular planning, conduit selection, and perioperative care have improved the safety and durability of this procedure, but continued research and collaboration are needed to refine management strategies and optimize long-term functional results.
